# Immobilization of DNA Aptamers on Polyester Cloth for Antigen Detection by Dot Blot Immunoenzymatic Assay (Aptablot)

**DOI:** 10.1155/2013/936542

**Published:** 2013-10-30

**Authors:** Sally Smiley, Maria DeRosa, Burton Blais

**Affiliations:** ^1^Ottawa Laboratory (Carling), Canadian Food Inspection Agency, Ottawa, ON, Canada; ^2^Department of Chemistry, Carleton University, Ottawa, ON, Canada

## Abstract

A simple dot blot immunoenzymatic assay system was developed using polyester cloth coated with an oligo-DNA aptamer to provide a high-affinity macroporous surface for the efficient capture of a model protein analyte (thrombin) in complex sample matrices such as foods. Bound thrombin was detected immunoenzymatically using a peroxidase-linked antithrombin antibody and a chromogenic substrate. A unique feature of this approach, which we have termed “aptablot,” is the facile immobilization of DNA aptamers on the polyester surface by cross-linking with a brief exposure to ultraviolet light, and the simple assay format obviating the need for specialized instruments. The assay principle described herein should be broadly applicable to many situations where analytes must be detected in complex samples, with the main limiting factor being the availability of suitable DNA aptamers.

## 1. Introduction

The detection of specific macromolecules (e.g., protein antigens) remains an important concern in the fields of human and animal health diagnostics, food safety testing, and basic research. One of the most widely used approaches for this purpose is the Enzyme Immunoassay (EIA) technique. The classic “sandwich” EIA utilizes specific antibodies immobilized on a solid phase to create a high-affinity surface for the capture of antigens, which are subsequently detected by reaction with an enzyme-linked antibody. The use of antibodies as assay reagents suffers from the high cost and time required for their production, as well as variability in stability, quality, and yield which may occur from one batch to another. Coating the solid phase with capture antibody consumes a high quantity of the available antibody, which must be applied in large excess to drive its immobilization on the solid phase. A variety of solid phases such as nonporous polystyrene microwells and microporous membranes lend themselves to the immobilization of antibodies for EIA procedures. Macroporous polyester cloth has also been used as a solid phase for the immobilization of antibodies in the development of simple dot blot assays [1]. Compared to nonporous and microporous solid phases, polyester cloth has the advantages of offering a readily available large surface for the immobilization of immunoreagents promoting rapid immunoreaction kinetics and ease of washing between reaction steps.

 Aptamers made of DNA or RNA oligonucleotides have emerged as a new class of synthetic receptors which in some applications may replace antibodies as reagents for the assay of macromolecule analytes, such as proteins associated with pathogenic bacteria [2]. Aptamers recognizing a wide array of different proteins have been produced, and there have been several reports of their adaptation as detection reagents in biosensors and other assay devices in which they are immobilized on a solid surface to serve in signal transduction upon binding with an analyte [3]. Many of these assay systems utilize specialized custom-built devices or complex chemistries for aptamer immobilization and reagent detection.

 Here, we examine the of use of polyester cloth as an adsorbent for DNA aptamers in the development of simple assays utilizing cloth strips upon which multiple samples can be blotted and hence processed simultaneously. The immobilization of oligo-DNA probes on polyester cloth by brief exposure to ultraviolet light for use in the detection of polymerase chain reaction (PCR) products by hybridization has previously been shown [4], and it is surmised presently that this simple approach should also permit the immobilization of DNA aptamers to create a high affinity capture surface for the assay of target analytes. In this “aptablot” approach, target molecules captured on the aptamer-coated cloth surface are detected by reaction with a dilute preparation of specific antibodies linked to a marker enzyme. The detection of thrombin is presently considered as a model system, since DNA aptamers for this protein have been well characterized [5, 6] and specific antithrombin antibodies are commercially available.

## 2. Experimental

### 2.1. Aptamers and Thrombin

Aptamers used in the present study were selected against the human coagulation protein thrombin (Table 1). All oligonucleotides were synthesized by Sigma Genosys (Oakville, ON, Canada). Aptamer 3 was designed for a different study by modifying Aptamer 2 to incorporate additional randomly generated sequences appended at the 5′ and 3′ ends for primer annealing for polymerase chain reaction applications (not covered in the present work). It was included in this study because it represents a version of thrombin-binding aptamer of intermediate size.

The human *α*-thrombin used in these studies was from a commercial source (Haematological Technologies Inc, HCT-0020, Essex Junction, VT) and was provided as a 8.9 mg/mL stock with a specific activity of 4493 U/mg and an extinction coefficient (*E*_tcm, 280 nm_^1%^ = 18.3).

### 2.2. Sample Matrices

Sample matrices included pasteurized 1% milk and beef bouillon (ingredients: beef broth (water, beef stock), yeast extract, salt, flavour, salted onion juice, caramel, disodium inosinate, and disodium guanylate), purchased from a local grocer, and the microbial growth medium brain heart infusion (BHI) broth (ingredients per litre: 12.5 g brain infusion solids, 5.0 g beef heart infusion solids, 10.0 g proteose peptone, 2.0 g glucose, 5.0 g sodium chloride, and 2.5 g disodium phosphate) (Oxoid CM1135, Ottawa, ON). 

### 2.3. Preparation of Aptamer-Coated Cloth by Spotting

Polyester cloth (DuPont Sontara 8100; DuPont, Mississauga, ON) cut into 2 × 5 cm strips and bearing a printed sample location grid with 5 cells was manufactured by contract with Dave's Custom Promotions, Inc. (Ottawa, Ontario, Canada), and washed with 95% (v/v) ethanol followed by rinsing with deionized distilled water on a filter with vacuum suction. The strips were air dried overnight prior to use for aptamer immobilization. 

DNA aptamers (Table 1) were prepared in high salt buffer (HSB: 0.1 M tris-HCl (pH 8.0), 0.01 M MgCl_2_, and 0.15 M NaCl) containing 30% (v/v) ethanol. Aptamer solution was applied in discrete spots (5 *μ*L) on the polyester cloth strips, followed by incubation for 3 hours at 37°C in a dry incubator. The aptamers were cross-linked to the dried strips by exposing the strips to UV light using a UV cross-linker set at 254 nm, 120 mJ cm^−2^ (Stratalinker model 1800; Stratagene, La Jolla, CA), and then washed five times with 0.01 M phosphate-buffered saline (pH 7.4), 0.85% (w/v) NaCl (PBS) containing 0.05% Tween 20 (PBST) on a filter under vacuum suction. The strips were blocked by incubation for 1 hour at 37°C with 0.5% (w/v) Bio-Rad protein blocking reagent (Bio-Rad 170-6404, Hercules, CA) in PBST, followed by washing with PBST.

### 2.4. Preparation of Aptamer-Coated Cloth by Saturation

Aptamer was prepared in HSB containing 30% (v/v) ethanol. Polyester cloth strips were saturated with 1 mL of aptamer solution and incubated for 3 hours at 37°C in a dry incubator. The aptamer was cross-linked to the dried strips by UV exposure as described above, then washed with PBST, and blocked. 

### 2.5. Thrombin Assay on Aptamer-Coated Cloth

The following reactions were carried out at room temperature in the dark. Cloth strips coated by spotting aptamer in discrete areas were reacted with thrombin by saturating with 1 mL of PBS containing 13.5 nM of human *α*-thrombin and incubating for 20 min. Strips coated by saturation of the entire surface with aptamer were reacted with thrombin samples by discretely spotting 5 *μ*L of human *α*-thrombin at various concentrations in different sample matrices (PBS, 1% milk, BHI broth, and beef bouillon) and then incubated for 20 min. Strips were then washed five times with PBST on a filter with vacuum suction. Bound thrombin was assayed by sequential 20 min reactions of the strips with 1 mL containing antihuman thrombin antibody (Haematological Technologies Inc, AHT-5020, Essex Junction, VT) diluted 1 : 1000 in PBST, and 1 mL of antimouse IgG-peroxidase conjugate (Sigma-Aldrich, A3673, Oakville, ON) diluted 1 : 1000 in PBST, with PBST washes after each step. Finally, the strips were saturated with 1 mL of TMB membrane peroxidase substrate (Kirkegaard & Perry Laboratory, 50-77-02, Gaithersburg, MD) and incubated for 10 minutes. Reactions were graded qualitatively as follows: positive (blue spot), negative (no spot).

## 3. Results and Discussion

### 3.1. Immobilization of Antithrombin Oligonucleotides

Initial experiments examined the immobilization and subsequent thrombin-capturing abilities of different aptamer sequences reported in the literature. For this purpose, oligonucleotides representing a range of sizes (15 to 105 nucleotides) and base sequences were immobilized in discrete spots on a strip of polyester cloth and then assessed for binding activity by flooding the entire strip with thrombin solution, followed by detecting bound thrombin by sequential reactions of the strip with antithrombin monoclonal antibody, antimouse IgG-peroxidase conjugate, and TMB substrate. In addition to the thrombin-binding sequences, a 50-mer oligonucleotide probe originally conceived for hybridization with a verotoxin (VT 1) PCR amplicon [4] was also immobilized on the cloth strip to serve as a control for thrombin binding. A concentration of 10 *μ*M for each oligonucleotide, previously shown to be satisfactory for the immobilization of capture probes in PCR applications [4], was used in these experiments.

**Figure 1:**
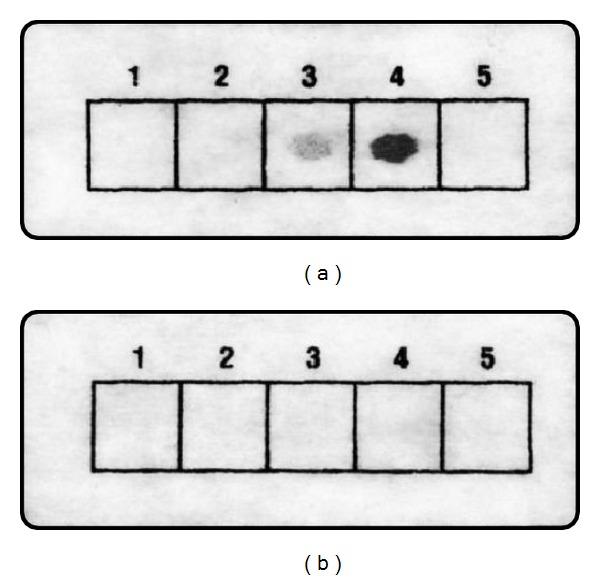
Effect of oligonucleotide sequence. Different oligonucleotides (1, Apt 1; 2, Apt 2; 3, Apt 3; 4, Apt 4; 5, VT 1) were spotted on polyester cloth. The strips were either UV cross-linked (a) or not (b) and then saturated with thrombin solution followed by immunoenzymatic assay of bound thrombin as described in Methods. These experiments were repeated in a separate trial with identical results (not shown).

 Shorter aptamer sequences of 15 and 29 nucleotides in length did not produce any visible reaction with thrombin, whereas the longer 67- and 105-mer aptamers cross-linked to the polyester cloth by ultraviolet light exhibited significant thrombin-binding activity manifested as localized immunoenzymatic reactivity on the strip where these probes had been spotted (Figure 1). No reactivity was observed with the verotoxin probe spot, confirming that the reactions observed with the aptamers were due to their specific thrombin-binding activities and neither was thrombin-binding activity for any of the aptamers in the absence of ultraviolet exposure, suggesting that cross-linking the oligonucleotides to the polyester surface is essential for stable immobilization of the aptamers. The lack of reactivity of the shorter aptamers may be due to an inability of small DNA molecules to bind to the polyester surface, or to a perturbation in their thrombin-binding conformation caused by the cross-linking reaction. The longer oligonucleotides contained SELEX primer-binding sequences at their 5′ and 3′ which are not involved in aptamer function *per se*, and thus, these appended sequences may serve to provide an immobilization link with the polyester surface sparing the conformational integrity of the aptamer portion.

**Figure 2:**
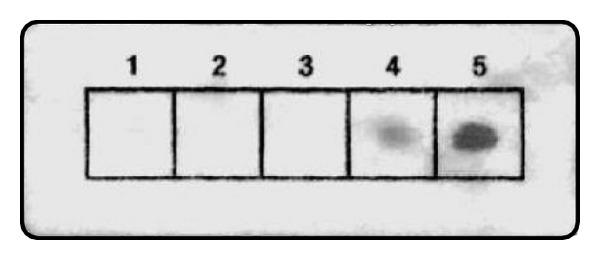
Effect of 5′-tailing of aptamer. Apt 2 was synthesized with different polynucleotide (*n* = 5) tails at the 5′ end (1, untailed Apt 2; 2, 5′-poly-A-tailed; 3, 5′-poly-C-tailed; 4, 5′-poly-G-tailed; 5, 5′-poly-T-tailed) and each was spotted on polyester cloth and then UV cross-linked as before. The strips were saturated with thrombin solution followed by immunoenzymatic assay of bound thrombin as described in Methods. These experiments were repeated in a separate trial with identical results (not shown).

Notwithstanding the foregoing, we investigated the possibility of enhancing the immobilization of shorter aptamers (e.g., the 29-mer aptamer) by synthesizing modified versions of Apt 2 bearing different short (*n* = 5) 5′-poly-nucleotide tails (i.e., poly-A, poly-C, poly-G, or poly-T) and immobilizing these on strips of polyester cloth by UV cross-linking as before. The ability of the immobilized aptamer to capture thrombin was determined by reacting the strips with thrombin solution, followed by immunoenzymatic assay of the bound thrombin. The addition of a poly-G tail produced a weak assay signal, but a more striking result was obtained with a poly-T tail, which produced a signal intensity equivalent to that observed with Apt 4 (Figure 2). These results suggest that poly-T tailing was particularly effective in enhancing the immobilization of the short aptamer in the correct form for analyte capture. The mechanism for enhanced immobilization of the short aptamer by poly-T-tailing may involve the activation of thymine reactivity by UV irradiation, which is known to occur when genomic DNA is damaged by UV radiation in living cells [7], or perhaps activation of double bonds or other active groups on the polyester (polyethylene terephthalate) surface. Whatever mechanism is involved in the immobilization of the DNA aptamers, these results clearly indicate that a suitable capture surface can be prepared using either a longer (e.g., Apt 4) or a 5′-poly-T-tailed (e.g., poly-T-Apt 2) aptamer sequence. Thus, in instances where operational or functional requirements dictate the use of a shorter oligonucleotide sequence, it is possible to achieve functional immobilization of smaller aptamers by the present poly-T-tailing approach, obviating the need for chemical derivatization or the incorporation of specialized nucleotides. However, since in the present model system there were no constraints on the length of oligonucleotide which could be used, and since Aptamer 4 exhibited strong assay signals (coloured spots), it was used for all subsequent experiments.

**Figure 3:**
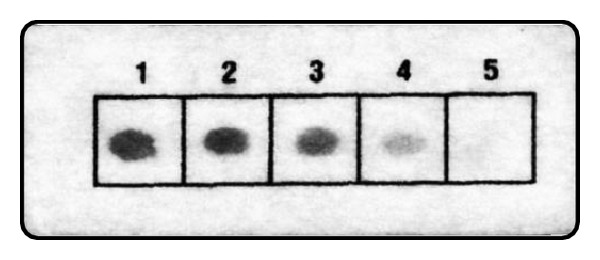
Effect of aptamer coating concentration. Apt 4 was spotted on polyester cloth at different concentrations (1, 2.00 *μ*M; 2, 1.00 *μ*M; 3, 0.50 *μ*M; 4, 0.25 *μ*M; 5, 0.12 *μ*M). After UV cross-linking, the strips were saturated with thrombin solution followed by immunoenzymatic assay of bound thrombin as described in Methods. These experiments were repeated in a separate trial with identical results (not shown).

The optimum concentration for coating the polyester cloth was determined by spotting different amounts of Apt 4 and then assaying using the immunoenzymatic procedure as before. A minimum of 0.25 *μ*M aptamer solution spotted on the strip produced a detectable assay signal, though the least concentration giving a strong signal intensity was 1 *μ*M (Figure 3), which was, therefore, used in subsequent experiments.

### 3.2. Detection of Thrombin in Different Sample Matrices

The preceding experiments utilized aptamer applied in localized spots on a strip of polyester cloth. The aptamer-thrombin interaction occurred in a defined buffer devoid of extraneous materials which might produce interference with the binding reaction. One foreseeable application of the aptablot system is the detection of a target analyte in different sample types, for example, allergens or pathogenic bacteria in various foods submitted for laboratory analysis. In such a scenario, it would be essential that the aptamer-target interaction be capable of proceeding in various sample matrix compositions, as might be encountered in food sample suspensions or enrichment broth cultures. Furthermore, the ideal assay format would utilize a cloth strip in which the entire surface is coated with the capturing agent, such that the strip could accommodate multiple test samples.

**Figure 4:**
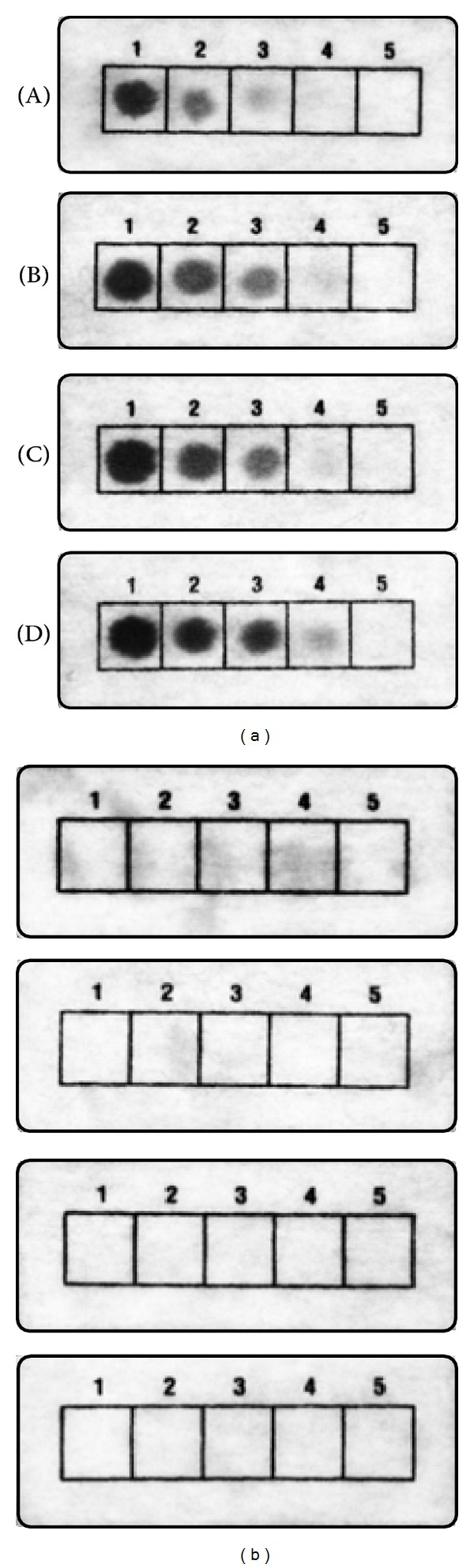
Effect of sample matrix. Thrombin was prepared at different concentrations (1, 13.50 nM; 2, 2.70 nM; 3, 1.35 nM; 4, 0.27 nM; 5, 0 nM) in a variety of matrices ((A) PBS, (B) 1% milk, (C) BHI broth, and (D) beef bouillon) and then spotted on polyester cloth strips with (a) and without (b) immobilized Apt 4, followed by immunoenzymatic assay of bound thrombin as described in Methods. These experiments were repeated in a separate trial with identical results (not shown).

To demonstrate how such a principle might work, strips of polyester cloth were entirely coated by saturation with Apt 4 solution. These were then used for the capture of thrombin from samples spotted in the cells of the printed grid. To determine the impact of the sample matrix, thrombin was diluted in different types of liquid samples containing complex mixtures of ingredients. The minimum quantity of thrombin producing a visible assay signal was 1.35 nM in PBS, 0.27 nM (with weak spot intensity) in 1% milk and BHI broth (a typical enrichment medium used in microbiological analyses of foods), and 0.27 nM (with stronger spot intensity) in beef bouillon (Figure 4). The reason for the slight improvement in thrombin detectability in the complex sample matrices is not known. The possibility that this might be attributable to the presence of elevated levels of salts which may affect the formation of active aptamer structures [8] was verified by repeating the assay of thrombin diluted in PBS containing 10 mM MgCl_2_ and 10 mM KCl, but the detectability in these buffers remained unchanged from that observed with PBS alone (not shown).

## 4. Conclusions

 We have demonstrated the applicability of a simple aptablot system using a DNA aptamer as an inexpensive synthetic capture agent for the sensitive assay of a protein analyte in complex samples. A unique feature of the aptablot system is the simple means by which the aptamer was immobilized on the polyester cloth surface using a brief exposure to ultraviolet light. Some flexibility in the choice of aptamer length is afforded by the observation that modification with a 5′-poly-T-tail enabled the efficient immobilization of a short sequence. While the present approach does not obviate the use of an antibody detector reagent, it does foster conservation of precious antibody stocks due to their application in highly diluted form, and it also enables the use of monoclonal detector antibodies in instances where the corresponding epitope on the target molecule is only present in one copy (which is likely the case in the detection of a monomeric protein such as thrombin). All of the components in the present assay system are readily available from commercial sources at reasonable cost. Furthermore, antibodies to many diagnostically relevant molecules are available from commercial sources, and it may be reasonably expected that the number of aptamer sequences to different analytes published in the scientific literature will grow at a great rate as this new reagent technology continues to evolve, expanding the scope of applications amenable to the aptablot approach. The *de novo* development of reagent aptamers to other analytes of interest using established protocols is not beyond the capabilities of most basic biochemistry laboratories, and one can envisage future applications in which even the detector antibody used in the present approach may be replaced with a second aptamer bearing an enzyme or other readily assayable marker. More work is needed to expand the scope of application of the aptablot approach from the present thrombin model system to the assay of diagnostically relevant analytes, such as microbial toxins, protein allergens, and environmental contaminants.

## Figures and Tables

**Table 1:** Oligonucleotide sequences used in this study.

Aptamer	Sequence (5′-3′)	Length (bp)	Source
Apt 1	GGTTGGTGTGGTTGG	15	[5]
Apt 2	AGTCCGTGGTAGGGCAGGTTGGGGTGACT	29	[6]
Apt 3	GCTCCTACAAATGCCATCATTAGTCCGTG	67	This study
	GTAGGGCAGGTTGGGGTGACTGCTGCAG		
	CGAGCTTACG		
Apt 4	AGATGCCTGTCGAGCATGCTCTTTGGAGA	105	[6]
	CAGTCCGTGGTAGGGCAGGTTGGGGTGA		
	CTTCGTGGAAGAAGCGAGACGGTGTAGC		
	TAAACTGCTTTGTCGACGGG		
VT 1	ACTGGATGATCTCAGTGGGCGTTCTTATG TAATGACTGCTGAA	50	[4]
